# An ensemble machine learning model based on magnetic resonance imaging features for diagnosing deep infiltrating endometriosis

**DOI:** 10.3389/fphys.2026.1753847

**Published:** 2026-05-29

**Authors:** Linlin Zhang, Xiaofeng Qing, Junying Zou, Hui Yang, Lanlan Yu

**Affiliations:** Department of General Gynecology, General Hospital of Hunan University of Medicine, Huaihua, Hunan, China

**Keywords:** AI-assisted diagnosis, deep infiltrating endometriosis, ensemble model, machine learning, magnetic resonance imaging, SHAP explanation

## Abstract

**Objective:**

To address the need for more objective and reproducible preoperative diagnosis of deep infiltrating endometriosis (DIE), this study aimed to develop and validate a weighted ensemble machine learning (ML) model using multimodal magnetic resonance imaging (MRI) and clinical features, and to compare its performance against radiologists.

**Methods:**

This retrospective study enrolled 330 patients (168 in DIE group, 162 in control group) who underwent MRI examinations and surgical-pathological confirmation during January 2020 to June 2025. Following feature selection, five ML models were constructed: logistic regression (LR), support vector machine (SVM), random forest (RF), XGBoost, and CatBoost. A weighted ensemble model was developed via soft voting, weighting each model’s prediction by its AUC on validation set. Model performance and clinical net benefit were assessed using ROC curves, calibration curves, and decision curve analysis. Model interpretability was achieved using a weighted SHapley additive exPlanations (SHAP) method. The ensemble model’s performance was compared to two radiologists’ independent readings.

**Results:**

The ensemble model demonstrated superior performance on the independent test set, achieving an AUC of 0.938 (95% CI: 0.883-0.979; sensitivity, 0.920; accuracy, 0.859), outperforming all individual models. Weighted SHAP analysis identified C-reactive protein, cancer antigen 125, T2-weighted signal intensity, and symptom duration as top contributors. The radiologists’ average AUC was 0.818 (κ = 0.42), whereas the ensemble model achieved a higher AUC on the same dataset (P = 0.002). With AI assistance, the radiologists’ accuracy increased to 0.920 and interpretation time decreased by approximately 30%.

**Conclusion:**

The weighted ensemble ML model, integrating multimodal MRI and clinical features, enables high-accuracy DIE identification with favorable stability and interpretability. This model holds significant potential as an effective clinical decision-support tool for radiologists.

## 1 Introduction

Endometriosis (EM) is a prevalent chronic gynecological disorder characterized by the presence and cyclic response of endometrial-like tissue outside the uterine cavity, often leading to chronic pelvic pain, infertility, and diminished quality of life (Koninckx et al., 2021). Epidemiological research indicates that approximately 10% of women of reproductive age are affected to varying degrees, with about 20%-30% of cases involving deep infiltrating EM (DIE) (Gruber et al., 2021). DIE is defined by lesions penetrating more than 5 mm beneath the peritoneum, frequently involving the uterosacral ligaments, rectovaginal septum, bladder, and ureters. Due to complex anatomy, occult lesions, and diverse imaging manifestations, its clinical diagnosis and preoperative assessment remain challenging.

Magnetic resonance imaging (MRI), with its high soft-tissue resolution and diverse imaging sequences, is the preferred imaging modality for evaluating DIE (Rousset et al., 2023). However, conventional MRI diagnosis heavily relies on the subjective experience of radiologists, leading to limited diagnostic accuracy and consistency, particularly for lesions with high signal heterogeneity and irregular morphology (Ibrahim et al., 2021). In recent years, radiomics combined with machine learning (ML) has emerged as a novel analytical approach, capable of automatically extracting vast quantities of quantitative features from medical images to identify underlying patterns, thereby enabling objective and reproducible disease diagnosis and prediction (Chen et al., 2023; Jin et al., 2024).

Building on this, ensemble ML, which amalgamates predictions from multiple algorithms to synthesize their respective strengths, can significantly enhance diagnostic stability and generalizability, especially with limited sample sizes. This methodology has shown considerable promise in oncology, neuroimaging, and gynecological diseases (Guo et al., 2023; Vos et al., 2023). Nevertheless, systematic research integrating MRI features with ensemble ML specifically for DIE remains scarce, and there is a lack of efficient and interpretable intelligent diagnostic models for this condition.

In recent years, radiomics and machine learning techniques have been increasingly applied in gynecologic imaging to extract quantitative imaging biomarkers beyond conventional visual assessment. Several studies have explored MRI-based radiomic signatures for the detection or characterization of endometriosis, suggesting that texture features may capture subtle tissue heterogeneity associated with ectopic endometrial infiltration (Lambin et al., 2012; Peluso et al., 2026). However, most existing studies are limited by relatively small sample sizes, single-model frameworks, or lack of integration between imaging and clinical biomarkers(Shrestha, Shrestha et al., 2025). Moreover, the application of ensemble machine learning strategies for improving diagnostic robustness in deep infiltrating endometriosis remains insufficiently investigated.

Therefore, this study aims to develop and validate an ensemble ML diagnostic model based on multi-sequence MRI features, to explore its value in the automated identification and auxiliary diagnosis of DIE, and to analyze the model’s key features and clinical interpretability, thereby offering a novel technical approach for preoperative DIE assessment.

## 2 Materials and methods

### 2.1 Study population

This single-center, retrospective study utilized data from patients who underwent pelvic MRI examinations and subsequent surgical-pathological confirmation at our hospital between January 2020 and June 2025. The study protocol strictly adhered to the principles of the Declaration of Helsinki and was approved by the Ethics Committee of General Hospital of Hunan University of Medicine.

Based on preliminary results and previous literature (Brunelli et al., 2023), the area under the curve (AUC) for conventional MRI diagnosis of DIE is approximately 0.75, while machine learning models have been reported to achieve AUC values around 0.85 in similar radiomics-based diagnostic studies. Using parameters of α = 0.05 and statistical power = 0.8, sample size estimation performed with MedCalc software indicated that a minimum of approximately 132 patients in the DIE group would be required. To reduce potential bias caused by class imbalance and to ensure stable machine learning model training, a relatively balanced distribution of cases and controls was used in the dataset, yielding a target sample size of approximately 260 patients. Considering potential exclusions due to image quality issues and missing clinical data, 168 patients with DIE and 162 control patients were ultimately included, resulting in 330 valid cases for the final analysis, which satisfied the statistical requirements for model development.

Inclusion criteria for DIE group (case group): (1) Surgical and pathological confirmation of DIE; (2) Standardized MRI examination performed within 4 weeks prior to surgery; (3) High image quality and complete clinical data. Exclusion criteria for DIE group: (1) Coexisting malignant tumors or severe pelvic infection; (2) Previous major surgery causing significant anatomical distortion; (3) Significant MRI artifacts or missing key sequences. Among the final DIE cases, locations included uterosacral ligaments (n = 56), rectovaginal septum (n = 38), bladder/ureter (n = 28), and other sites (n = 32). All patients were women of reproductive age.

Inclusion criteria for control group: (1) Symptomatic controls: Patients with pelvic pain/dysmenorrhea and initial clinical suspicion of DIE, but confirmed negative for DIE by surgical pathology or ≥ 12 months of follow-up; (2) Non-DIE endometriosis controls: Patients with surgical-pathological confirmation of non-DIE endometriosis (ovarian endometriomas or peritoneal endometriosis). Cases and controls were frequency-matched for age (± 3 years), body mass index (BMI, ± 2 kg/m²), and examination year. Common exclusion criteria (for both groups): Coexisting malignant tumors, history of major pelvic surgery causing severe anatomical alteration, poor MRI image quality, or absence of crucial sequences.

### 2.2 MRI acquisition and preprocessing

All pelvic MRI examinations were performed using 3.0-T scanners (Siemens Skyra or GE Discovery MR750) with a standardized pelvic imaging protocol including T1-weighted imaging (T1WI), T2-weighted imaging (T2WI), and diffusion-weighted imaging (DWI). In selected cases, contrast-enhanced T1WI (CE-T1WI) was also obtained. Patients were instructed to empty the bladder and maintain moderate filling to reduce motion and susceptibility artifacts.

Image quality was independently evaluated by two experienced radiologic technologists using a five-point scale, and images with scores <3 were excluded. All qualified images were exported in DICOM format and underwent standardized preprocessing including N4 bias field correction, isotropic resampling (1 × 1 × 1 mm³), and grayscale intensity normalization to reduce scanner-related variability and improve feature reproducibility, as recommended in radiomics methodological guidelines (Zwanenburg et al., 2020).

Radiomic features were extracted using a whole-image strategy rather than lesion-based manual segmentation. This approach was adopted to reduce potential inter-observer variability associated with manual ROI delineation and to capture global imaging characteristics that may reflect the complex and multifocal distribution of deep infiltrating endometriosis within the pelvis. In total, 41 candidate quantitative features were obtained, including first-order intensity features, texture features derived from gray-level matrices, and clinical variables. Shape-based features were not included because explicit lesion segmentation was not performed.

### 2.3 Lesion annotation

Considering the complex morphology, blurred boundaries, and multi-focal distribution of DIE lesions, this study adopted a whole-image radiomics strategy instead of manual region-of-interest (ROI) delineation. Following spatial resampling, intensity normalization, and N4 bias field correction, T1WI, T2WI, and DWI sequences were imported into the PyRadiomics (v3.1.0) platform for feature extraction. The extracted radiomics features encompassed four categories: (1) first-order statistical features; (2) shape-based features; (3) texture features [gray level co-occurrence matrix (GLCM), gray level run length matrix (GLRLM), gray level size zone matrix (GLSZM), neighboring gray tone difference matrix (NGTDM), gray level dependence matrix (GLDM)]; (4) filter-derived features [wavelet and Laplacian of Gaussian (LoG)]. To ensure feature stability, features with low variance and intraclass correlation coefficient (ICC) < 0.75 were excluded. The remaining features were standardized using z-score normalization before subsequent feature selection and modeling.

### 2.4 Feature selection and ensemble ML model development

Using the DIE versus control group classification as the target variable, the dataset was first randomly divided into a training set (70%) and an independent test set (30%) to avoid potential information leakage. All subsequent feature selection procedures were performed exclusively on the training dataset, while the independent test set was reserved only for the final model evaluation.

Within the training dataset, radiomics features initially underwent Spearman correlation analysis to remove highly collinear variables (|r| > 0.9). Univariate analysis was then performed, retaining features with significant differences between the two groups (P < 0.05). Given the exploratory nature of this machine learning modelling framework, formal multiplicity correction was not applied during the feature screening stage. Instead, dimensionality reduction and regularization were implemented using LASSO regression with cross-validation to reduce the risk of overfitting and false discovery. Subsequently, least absolute shrinkage and selection operator (LASSO) regression was employed for dimensionality reduction, with the optimal penalty parameter λ determined via ten-fold cross-validation, selecting features with the highest predictive value.

Clinical variables were incorporated together with radiomic features to construct the integrated predictive model. Continuous clinical variables (e.g., laboratory biomarkers) were standardized using z-score normalization to ensure comparable scales across features. Categorical variables were transformed using one-hot encoding to allow their inclusion in machine learning algorithms. After preprocessing, clinical and radiomic features were concatenated into a unified feature matrix, which served as the input for subsequent feature selection and model training procedures.

Based on the optimized feature set, five base ML models were constructed: logistic regression (LR), support vector machine (SVM), random forest (RF), extreme gradient boosting (XGBoost), and categorical boosting (CatBoost). Hyperparameters were optimized within the training set via five-fold cross-validation and grid search, aiming to maximize AUC, with an early stopping mechanism implemented to prevent overfitting. To address potential class imbalance during model training, class weights were incorporated into the loss functions of the algorithms using inverse-frequency weighting based on class distribution (DIE:control = 168:162), resulting in approximately balanced class weights during model training.

An ensemble ML model was then constructed using a soft voting strategy, where the final output score was obtained by a weighted average of the predicted probabilities from the base models. Weights were automatically assigned based on each model’s AUC performance on the validation set. The weights of the base models were determined according to their validation AUC values, allowing models with higher discriminative ability to contribute more to the final prediction. This AUC-weighted soft voting strategy provides a simple and robust ensemble approach that reduces the risk of overfitting in relatively small datasets and enhances model interpretability compared with more complex ensemble frameworks.

Finally, the performance of the ensemble model was compared with that of the individual base models using the independent test set to evaluate the generalization ability of the models. This whole-image radiomics strategy has also been reported in previous radiomics studies aiming to capture global imaging patterns when lesion boundaries are difficult to delineate precisely (Rizzo et al., 2018).

### 2.5 Radiologist comparison and AI-assisted reading

To evaluate the clinical applicability of the proposed model, two board-certified radiologists independently reviewed the MRI examinations in the independent test set. Radiologist A had 12 years of experience in pelvic MRI interpretation, and Radiologist B had 9 years of experience.

All cases were anonymized and presented in a randomized order. The radiologists were blinded to the pathological results and model predictions during the initial assessment. The interpretation was performed using a standard clinical workstation (RadiAnt DICOM Viewer, version 3.2) under routine diagnostic display conditions. Only imaging data were provided during the independent reading phase, and no additional clinical information was available.

After completing the independent reading session, a washout period of two weeks was applied before the AI-assisted reading phase. During the second session, the model predictions were provided as decision-support information, and the radiologists reassessed the same cases.

### 2.6 Model evaluation and performance metrics

A multi-dimensional evaluation framework was adopted to comprehensively assess model diagnostic performance. Classification performance was evaluated using accuracy, sensitivity, specificity, positive predictive value (PPV), negative predictive value (NPV), F1-score, and the AUC of the receiver operating characteristic (ROC) curve. Differences in performance between models were compared using DeLong’s test for AUC (*P* < 0.05 considered statistically significant). Additionally, calibration curves were plotted and the Brier score was calculated to assess the goodness-of-fit of predicted probabilities. To further validate clinical potential, decision curve analysis (DCA) was used to evaluate the net benefit across different decision thresholds. Model calibration was evaluated using calibration curves together with Brier scores and calibration slope and intercept to quantify the agreement between predicted probabilities and observed outcomes.

To assess agreement between model and human diagnosis, the ensemble model’s predictions were compared against the independent readings of two senior radiologists, calculating the Kappa coefficient of agreement (Kappa ≥ 0.75 indicating good agreement). Simultaneously, to enhance model interpretability, the SHapley additive exPlanations (SHAP) algorithm was applied to visualize and rank the importance of each feature, exploring the contribution of key MRI features to the model’s decisions and their potential pathological significance.

### 2.7 Statistical analysis

All statistical analyses were performed in Python (v3.10) and R (v4.2.3) environments. Python was primarily used for image feature extraction, ML modeling, and interpretability analysis (employing libraries such as Scikit-learn, XGBoost, SHAP), while R was used for traditional statistical analysis and model performance comparisons. The normality of continuous variables was assessed using the Shapiro-Wilk test. Normally distributed data were presented as mean ± standard deviation (x¯ ± s), and intergroup comparisons were made using independent samples *t*-tests. Non-normally distributed data were expressed as median (interquartile range) [M (P25, P75)] and compared employing the Mann-Whitney U test. Categorical variables were presented as n (%), and intergroup comparisons were conducted utilizing the Chi-squared (*χ²*) test or Fisher’s exact test. To explore independent correlations between clinical variables and DIE occurrence, multivariate logistic regression analysis was performed, calculating odds ratios (ORs) and their 95% confidence intervals (CIs). Model diagnostic performance was reported using AUC, accuracy, sensitivity, specificity, F1-score, PPV, and NPV. Differences in AUC between models were compared using DeLong’s test. Model stability was assessed using the Bootstrap method (1,000 repetitions) to estimate 95% CIs. Model calibration was evaluated via the Brier score and calibration curves. Clinical net benefit was assessed using DCA. All statistical tests were two-sided, and *P* < 0.05 was considered statistically significant.

## 3 Results

### 3.1 Overall study characteristics and clinical data comparison

A total of 330 subjects were enrolled, comprising 168 in the DIE group and 162 in the control group. No significant difference was observed in age between the two groups (*P* = 0.631), indicating well-matched baselines. BMI was slightly higher in the DIE group (*P* = 0.034). Symptom duration was significantly longer (*P* < 0.001), cancer antigen 125 (CA125) levels were markedly elevated (*P* < 0.001), hemoglobin levels were lower (*P* < 0.001), and C-reactive protein (CRP) was also significantly higher (*P* < 0.001) in the DIE group. Regarding clinical symptoms, the incidences of pelvic pain, dysmenorrhea, dyspareunia, infertility, and cyclical rectal pain were all significantly higher (all *P* < 0.001), and irregular menstruation was more common in the DIE group (*P* = 0.015). MRI image quality scores did not differ significantly between both groups (*P* = 0.750), meeting the requirements for image analysis. T2 signal intensity scores and DWI restriction scores were significantly higher in the DIE group (both *P* < 0.001). The average maximum lesion diameter in the DIE group was 28.42 ± 7.75 mm, volume was 2.90 ± 1.28 mL, and lesions predominantly showed heterogeneous enhancement (*P* = 0.036). Furthermore, the proportions of patients with coexisting adenomyosis and ovarian endometriomas were significantly higher in the DIE group (both *P* < 0.001) (**Table 1**). In summary, the two groups were well-matched in general demographic characteristics but showed significant differences in laboratory markers, symptomatic manifestations, and MRI features, providing a stable foundation for subsequent radiomics modeling.

**Table 1:** Comparison of baseline characteristics between DIE and control groups.

Category	Variable	DIE group (n = 168)	Control group (n = 162)	*Z/t/χ²*	*P*
General characteristics	Age (years)	35.00 (30.75-40.00)	36.00 (32.00-40.00)	-0.48	0.631
	BMI (kg/m²)	22.74 (20.73-25.26)	22.13 (19.82-24.56)	2.12	0.034
	Symptom duration (months)	29.85 ± 14.42	20.87 ± 11.04	6.37	< 0.001
Laboratory tests	CA125 (U/mL)	79.63 (53.72-114.59)	40.30 (27.99-61.13)	8.73	<0.001
	Hemoglobin (g/L)	117.27 ± 12.47	122.20 ± 12.32	-3.61	< 0.001
	CRP (mg/L)	9.74 ± 3.46	5.97 ± 1.99	12.16	< 0.001
Symptom presentation	Pelvic pain			24.83	< 0.001
	Yes	113 (67.3%)	48 (29.6%)		
	No	55 (32.7%)	114 (70.4%)		
	Dysmenorrhea			11.51	< 0.001
	Yes	117 (69.6%)	51 (31.5%)		
	No	51 (30.4%)	111 (68.5%)		
	Dyspareunia			4.34	< 0.001
	Yes	114 (67.9%)	53 (32.7%)		
	No	54 (32.1%)	109 (67.3%)		
	Infertility			4.46	< 0.001
	Yes	117 (69.6%)	55 (34.0%)		
	No	51 (30.4%)	107 (66.0%)		
	Regular menstruation			0.48	0.015
	Regular	128 (76.2%)	141 (87.0%)		
	Irregular	40 (23.8%)	21 (13.0%)		
	Cyclical rectal pain			4.01	< 0.001
	Yes	71 (42.3%)	25 (15.4%)		
	No	97 (57.7%)	137 (84.6%)		
	Cyclical bladder pain			1.54	0.171
	Yes	30 (17.9%)	20 (12.3%)		
	No	138 (82.1%)	142 (87.7%)		
MRI features	MRI image quality score	4.45 (4.10-4.76)	4.39 (4.07-4.82)	0.32	0.750
	T2 signal intensity score	2.60 (2.40-2.80)	2.05 (1.80-2.20)	6.22	< 0.001
	DWI restriction score	2.40 (2.10-2.70)	1.75 (1.40-2.00)	6.11	< 0.001
	Maximum lesion diameter (mm)	28.42 ± 7.75	18.22 ± 5.83	13.55	< 0.001
	Lesion volume (mL)	2.90 ± 1.28	1.54 ± 0.73	11.83	< 0.001
	Enhancement pattern			6.63	0.036
	Heterogeneous	90 (53.6%)	64 (39.5%)		
	Homogeneous	55 (32.7%)	71 (43.8%)		
	None	23 (13.7%)	27 (16.7%)		
Concomitant lesions	Adenomyosis			7.04	< 0.001
	Yes	130 (77.4%)	53 (32.7%)		
	No	38 (22.6%)	109 (67.3%)		
	Ovarian endometrioma			5.04	< 0.001
	Yes	112 (66.7%)	46 (28.4%)		
	No	56 (33.3%)	116 (71.6%)		

DIE, Deep infiltrating endometriosis; BMI, Body mass index; CA125, Cancer antigen 125; CRP, C-reactive protein; MRI, Magnetic resonance imaging; DWI, Diffusion-weighted imaging.

**Figure 1:**
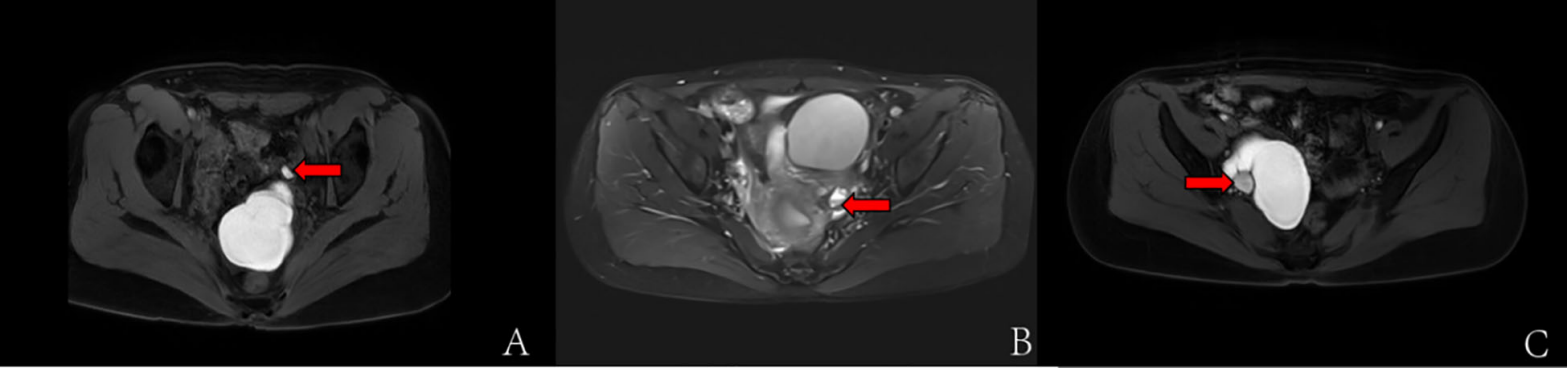
Representative MRI findings in DIE patients. **(A)** Female, 40 years old, post-operative left ovarian endometrioma, with multiple deep infiltrating lesions visible on the surface of the bowel and omentum (arrow). **(B)** Female, 38 years old, left ovarian endometrioma, with multiple patchy lesions visible on the posterior uterine wall and peritoneal surface (arrow). **(C)** Female, 29 years old, bilateral ovarian endometriomas, with multiple pelvic lesions involving the anterior rectal space (arrow). MRI, Magnetic resonance imaging; DIE, Deep infiltrating endometriosis.

Additionally, to visually represent the MRI characteristics of DIE, this study selected images from three representative typical cases (**Figure 1**). Lesions were predominantly located in the uterosacral ligaments, ovaries, and surrounding bowel, manifesting as heterogeneous hyperintensity on T2WI and marked diffusion restriction on DWI. Some lesions exhibited blurred margins, irregular morphology, accompanied by peripheral fibrosis and small cystic changes. These imaging findings are consistent with the high-weight features identified by the model (T2 signal intensity score, DWI restriction score, lesion volume, etc.), providing an imaging basis for the ML model.

### 3.2 Feature extraction and selection results

**Figure 2:**
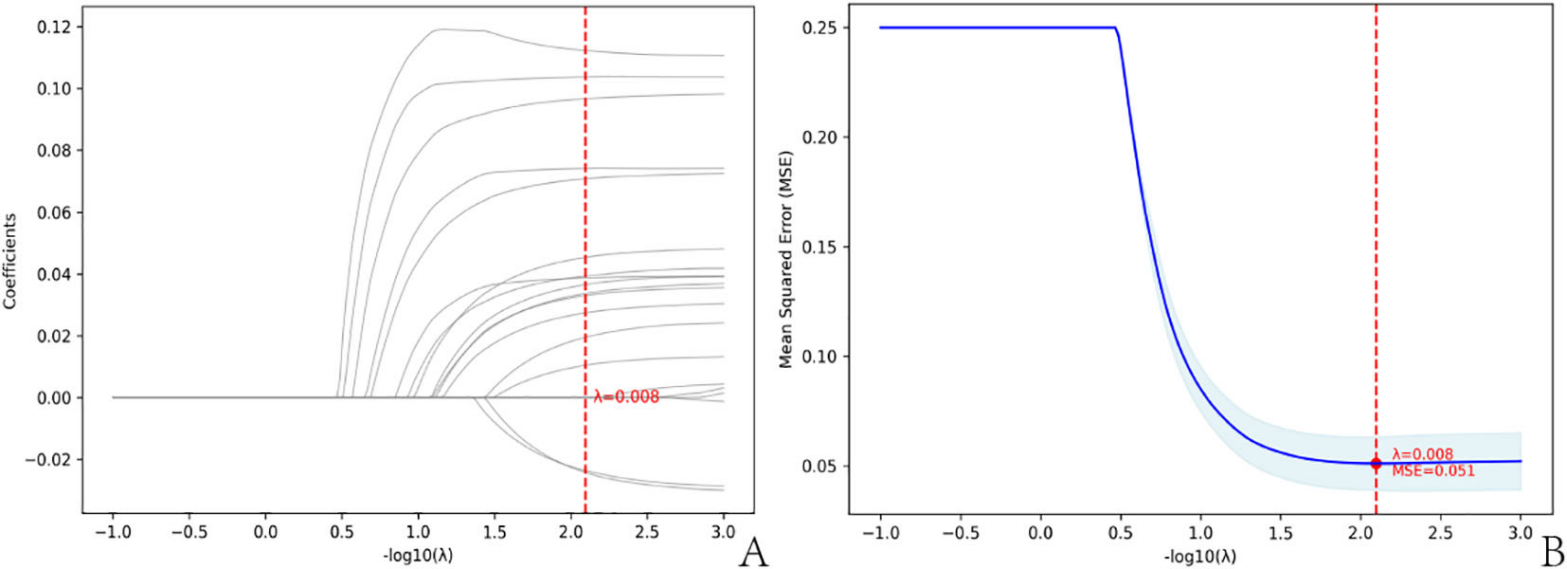
LASSO regression feature selection results. **(A)** LASSO path plot (red dashed line indicates optimal *λ* = 0.008); **(B)** Ten-fold cross-validation error curve (annotated with λ and minimum MSE point). LASSO, Least absolute shrinkage and selection operator; MSE, mean squared error.

A total of 41 candidate quantitative features were initially obtained from the MRI images and clinical data after standardized preprocessing. To avoid potential information leakage, the dataset was first randomly divided into a training set (70%, n = 231) and an independent test set (30%, n = 99). Because the numbers of DIE and control cases were relatively balanced (168 vs. 162), no additional class weighting or oversampling strategies were applied during model training. All feature filtering and selection procedures were performed exclusively within the training set, while the test set was reserved solely for final model evaluation. First, Spearman correlation analysis was applied within the training set to identify highly correlated variables (|r| > 0.7), and redundant features were removed to reduce multicollinearity. Subsequently, univariate statistical testing (Mann–Whitney U test) was performed on the remaining variables to identify features significantly associated with the presence of deep infiltrating endometriosis (P < 0.05). Finally, least absolute shrinkage and selection operator (LASSO) regression with 10-fold cross-validation was applied within the training set to further reduce dimensionality and identify the most predictive features. The LASSO coefficient profiles and cross-validation results are presented in **Figure 2A** and **Figure 2B**. The optimal penalty parameter was determined using the minimum mean squared error criterion (λ = 0.008, MSE = 0.0511).

Based on this procedure, seven key features were retained for subsequent model development. These selected features included ADC mean, lesion maximum diameter, T2 signal intensity score, CA125 level, age, BMI, and uterine retroversion status, which were then used as input variables for machine learning model construction. The detailed feature selection results are summarized in **Table 2**.

**Table 2:** Key features retained after LASSO selection.

Rank	Feature name	LASSO coefficient
1	T2_signal_intensity_score	0.112
2	DWI_restriction_score	0.104
3	Lesion_diameter_mm	0.097
4	CRP_mgL	0.074
5	Lesion_volume_ml	0.071
6	Dysmenorrhea	0.045
7	Adenomyosis	0.039
8	CA125	0.039
9	Symptom_months	0.037

LASSO, Least absolute shrinkage and selection operator; DWI, Diffusion-weighted imaging; CRP, C-reactive protein; CA125, Cancer antigen 125.

### 3.3 Diagnostic performance comparison of individual models

Given that the features selected by LASSO regression were predominantly explicit imaging indicators, to verify the independent discriminatory value of basic clinical and MRI acquisition parameters in DIE identification, five individual ML models (LR, SVM, RF, XGBoost, CatBoost) were further constructed. After excluding qualitative lesion features (T2 signal, DWI restriction score, lesion dimensions and volume, enhancement pattern, etc.) and obviously correlated clinical variables, 14 basic indicators were retained (age, BMI, symptom months, CA125, MRI_quality_score, T1WI, T2WI, DWI, CE_T1WI, gravida, para, c_section_history, menstrual_regular, contraception use) for model training. Performance was evaluated after validation on the 30% independent test set. The results showed (**Table 3**) that the five models achieved AUCs ranging from 0.900 to 0.936 on the test set, all demonstrating excellent discriminatory ability. Among them, the RF and CatBoost models achieved the highest AUCs (both 0.936; 95% CI: 0.884-0.973 and 0.883-0.976, respectively), suggesting an advantage of ensemble methods in recognizing non-linear features. The XGBoost model had the highest sensitivity (0.920), making it the most sensitive for DIE detection. SVM and RF showed relatively higher specificity (approximately 0.796), indicating greater robustness in negative identification. Overall accuracy ranged from 0.788 to 0.869, and F1-scores from 0.807 to 0.876, indicating balanced classification performance across models.

**Table 3:** Diagnostic performance comparison of individual ML models on the test set.

Model	AUC (95% CI)	Sensitivity	Specificity	Accuracy	F1-score
LR	0.903 (0.834-0.954)	0.880	0.694	0.788	0.807
SVM	0.900 (0.831-0.949)	0.860	0.796	0.828	0.835
RF	0.936 (0.884-0.973)	0.900	0.796	0.848	0.857
XGBoost	0.930 (0.869-0.976)	0.920	0.776	0.848	0.860
CatBoost	0.936 (0.883-0.976)	0.920	0.816	0.869	0.876

ML, Machine learning; LR, Logistic regression; SVM, Support vector machine; RF, Random forest; XGBoost, Extreme gradient boosting; CatBoost, Categorical boosting; AUC, Area under the curve; CI, Confidence interval.

Comprehensive analysis revealed that the CatBoost model performed best in terms of AUC, sensitivity, accuracy, and F1-score (AUC = 0.936, sensitivity = 0.920, F1-score = 0.876), maintaining high sensitivity while demonstrating good specificity (0.816), reflecting excellent robustness and generalizability.

**Figure 3:**
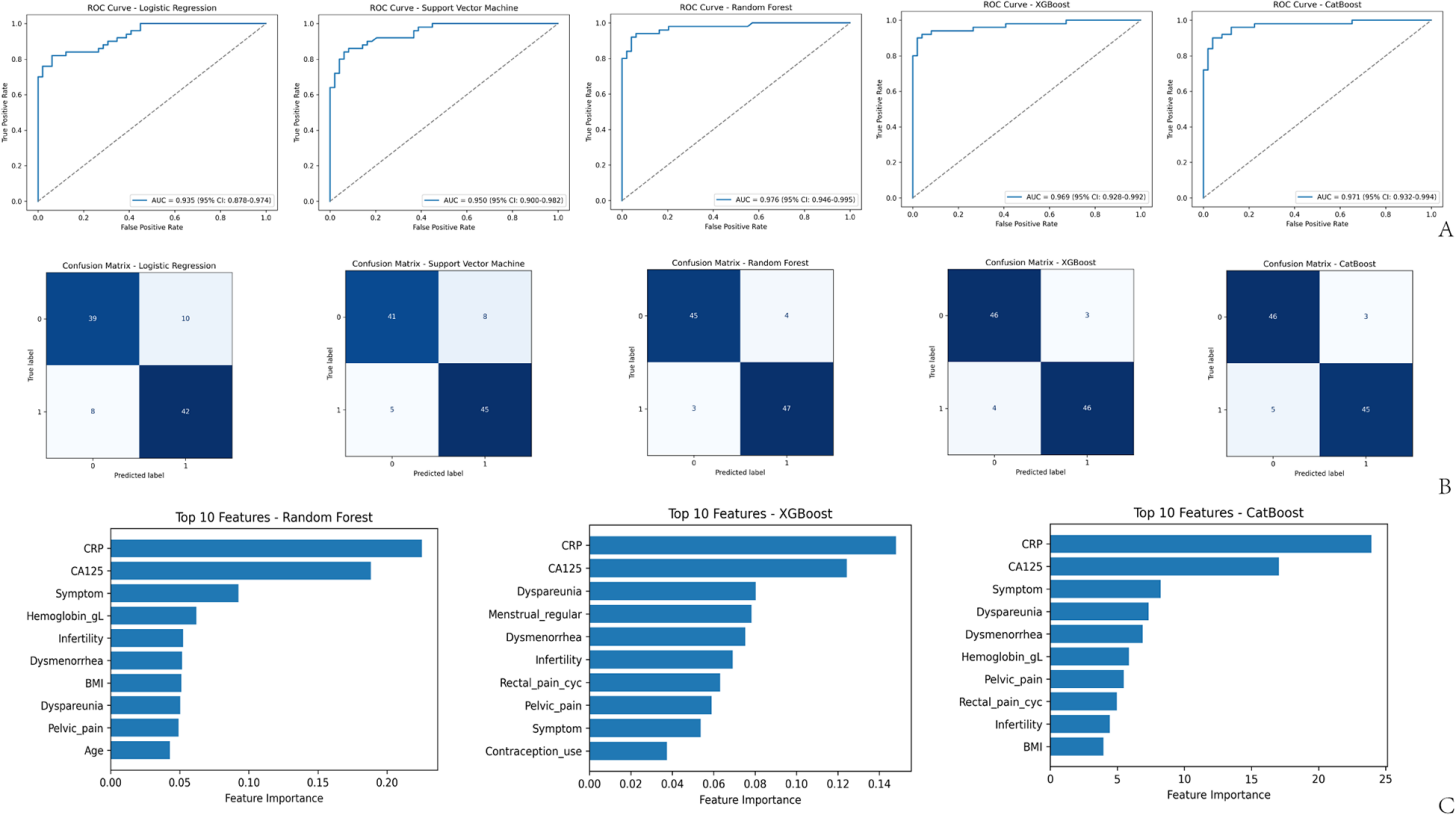
Diagnostic performance comparison of individual ML models. **(A)** ROC curves of the five models; **(B)** Confusion matrix comparison for the five models; **(C)** Feature importance analysis for RF, XGBoost, and CatBoost models. ML, Machine learning; ROC, Receiver operating characteristic; XGBoost, Extreme gradient boosting; CatBoost, Categorical boosting.

Furthermore, the CatBoost model’s feature importance analysis identified T2 signal intensity score, DWI restriction score, maximum lesion diameter, lesion volume, CRP, and CA125 as the main contributing variables, highly consistent with the key features identified in the prior LASSO selection, validating their clinical relevance and biological plausibility (**Figure 3**).

### 3.4 Ensemble ML model performance and stability validation

To further enhance discriminant accuracy and robustness, a weighted ensemble model was constructed based on the three best-performing individual models: RF, XGBoost, and CatBoost. On the independent test set, the ensemble model’s overall performance surpassed that of the individual models (**Table 4**). The ensemble model achieved an AUC of 0.938 (95% CI: 0.883-0.979), slightly higher than RF (0.936) and CatBoost (0.936), and superior to XGBoost (0.930). Its sensitivity was 0.920, matching CatBoost, the F1-score was 0.868, and accuracy was 0.859, indicating balanced classification capability. Although its specificity (0.796) was slightly lower than CatBoost’s (0.816), its overall composite performance was optimal, suggesting that the weighted ensemble strategy effectively balanced sensitivity and specificity across different models, enhancing stability and generalizability.

**Table 4:** Performance comparison between ensemble model and individual models.

Model	AUC (95% CI)	Sensitivity	Specificity	Accuracy	F1-score	Brier score	Calibration slope	Calibration intercept
RF	0.936 (0.881-0.979)	0.900	0.796	0.848	0.857	0.142	0.91	0.05
XGBoost	0.930 (0.869-0.977)	0.920	0.776	0.848	0.860	0.136	0.93	0.04
CatBoost	0.936 (0.880-0.978)	0.920	0.816	0.869	0.876	0.131	0.95	0.03
Ensemble	0.938 (0.883-0.979)	0.920	0.796	0.859	0.868	0.128	0.97	0.02

RF, Random forest, XGBoost, Extreme gradient boosting, CatBoost, Categorical boosting, AUC, Area under the curve, CI, Confidence interval.

**Figure 4:**
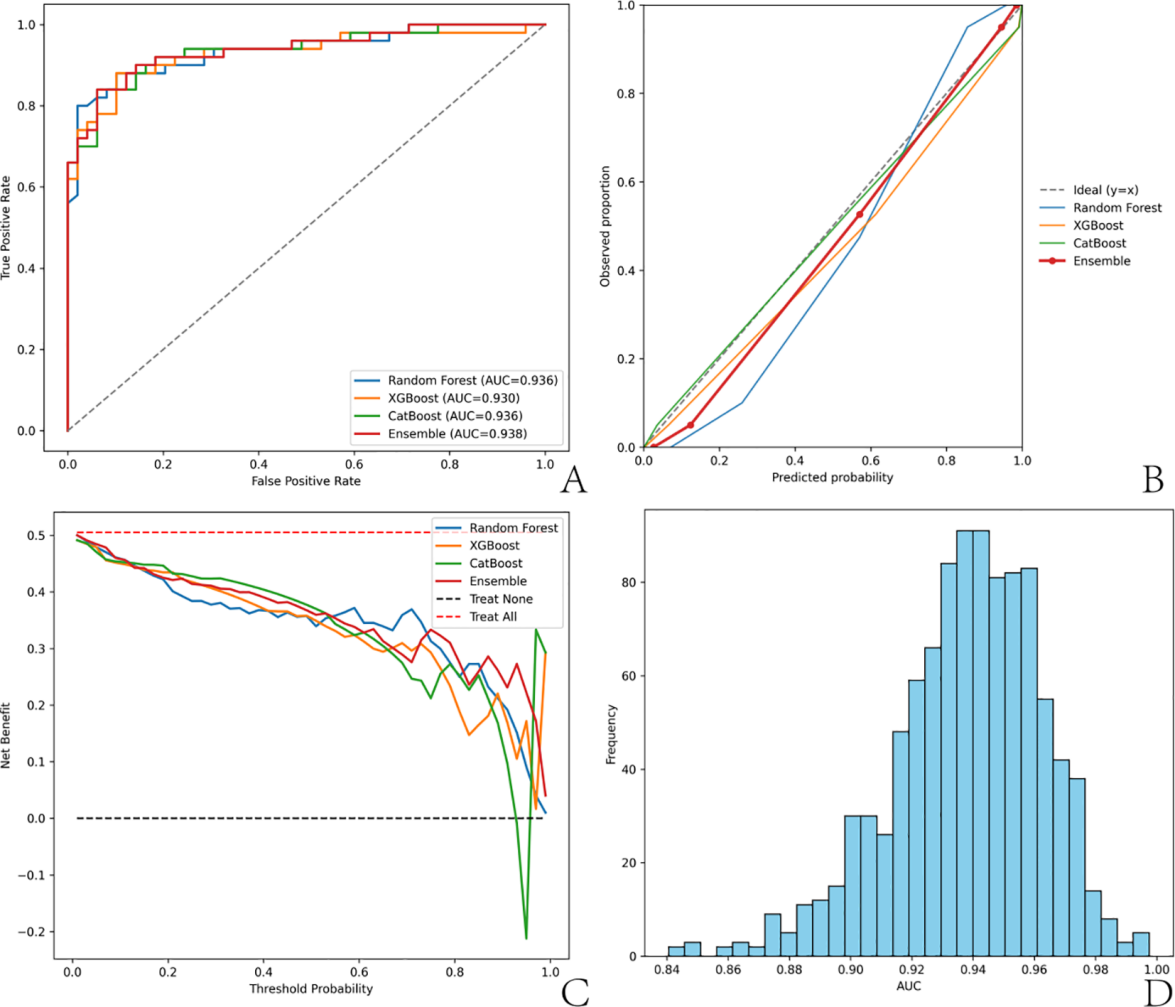
Ensemble ML model performance and stability validation. **(A)** ROC curve comparison between the ensemble model and the three individual models; **(B)** Multi-model calibration curves showing the agreement between predicted probability and actual occurrence (grey dashed line represents the ideal calibration line); **(C)** Multi-model DCA evaluating net benefit across different thresholds; **(D)** Histogram of AUC distribution from 1,000 Bootstrap resampling iterations for model stability validation. ML, Machine learning; ROC, Receiver operating characteristic; DCA, Decision curve analysis; AUC, Area under the curve.

ROC curve analysis (**Figure 4A**) showed that the ensemble model’s curve lay above those of the individual models, indicating higher discriminatory efficacy. In the multi-model calibration plot (**Figure 4B**), the ensemble model’s predicted probabilities most closely approximated the ideal calibration line (y = x), indicating the best agreement between predicted probabilities and observed outcomes. Quantitative calibration metrics further supported this finding (**Table 4**): the ensemble model showed the lowest Brier score (0.128) with a calibration slope of 0.97 and an intercept of 0.02, indicating good probability calibration. For comparison, the RF model yielded a Brier score of 0.142 (slope 0.91, intercept 0.05), the XGBoost model yielded a Brier score of 0.136 (slope 0.93, intercept 0.04), and the CatBoost model yielded a Brier score of 0.131 (slope 0.95, intercept 0.03). DCA (**Figure 4C**) further revealed that the ensemble model provided the highest net benefit across the threshold probability range of 0.2-0.8, suggesting optimal clinical utility for most decision thresholds. The AUC distribution obtained from 1,000 Bootstrap resampling iterations (**Figure 4D**) showed a concentrated trend with small variance, suggesting relatively stable results under bootstrap resampling within the current dataset.

### 3.5 Interpretability analysis based on weighted SHAP fusion

To deeply elucidate the decision logic and feature contributions of the ensemble model, this study employed an AUC-weighted SHAP fusion method for model interpretability analysis. This method integrated the SHAP values from the three sub-models (RF, XGBoost, CatBoost) using a weighted average (weight ratio: 0.334, 0.332, 0.334, respectively, based on their test set performance), thereby obtaining a unified feature importance interpretation for the ensemble model.

**Figure 5:**
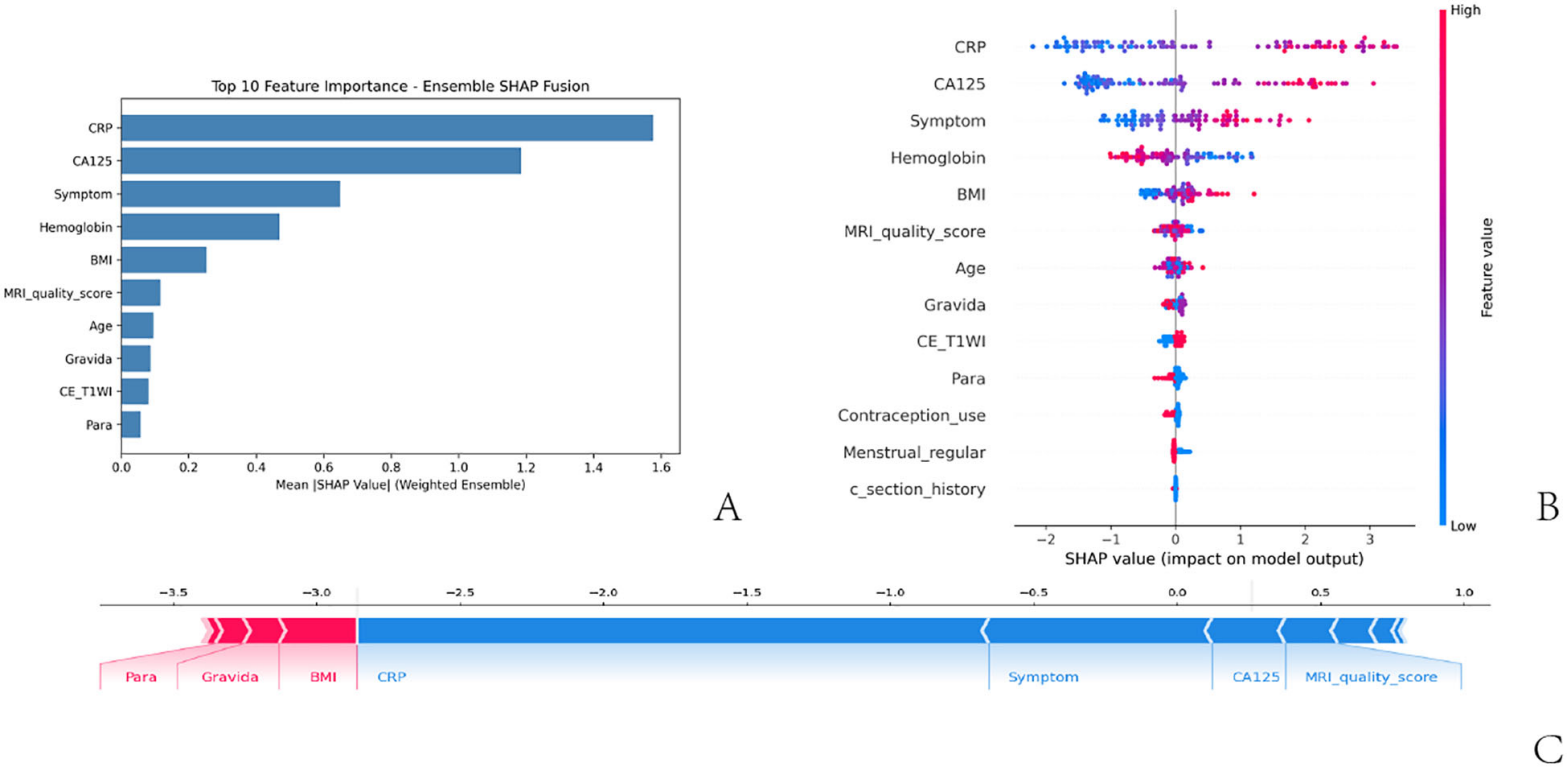
Weighted SHAP interpretability analysis of the ensemble model. **(A)** Global feature importance plot for the ensemble model; **(B)** Global SHAP summary plot; **(C)** Local SHAP force plot for an individual instance. SHAP, SHapley additive exPlanations.

In the global feature importance ranking (**Figure 5A**), CRP and CA125 contributed most to the model’s predictions, indicating the dominant role of inflammatory response levels and tumor markers in DIE identification. These were followed by symptom duration, hemoglobin, and BMI, suggesting that clinical inflammatory status, bleeding manifestations, and nutritional status also hold significant weight in distinguishing DIE from non-DIE individuals. Features like MRI quality score and age had smaller contributions but still reflected the potential influence of image acquisition consistency and patient physiological status on model discrimination.

The global SHAP summary plot (**Figure 5B**) further reveals the directional impact of feature values on the prediction outcome: high values of CRP and CA125 clearly pushed the model output towards a DIE-positive prediction (positive SHAP value), whereas higher hemoglobin and BMI were associated with non-DIE predictions (negative SHAP value). Additionally, the symptom months variable exerted a positive effect, indicating that as symptom duration or severity increases, the model leans more towards a DIE diagnosis. Overall, the directional effects of features in the ensemble model align with clinical pathological mechanisms, enhancing its interpretability and medical credibility.

At the individual level (**Figure 5C**), the SHAP force plot illustrates the combined directional influence of features on the prediction for a single patient. It showed that high values of CRP, CA125, and symptom duration collectively drove the model output towards a positive prediction, while relatively high BMI and hemoglobin values inhibited a shift towards a positive prediction, reflecting the dominant role of this individual’s clinical inflammatory and metabolic characteristics in the final discrimination.

To further explore the relative contributions of clinical and imaging variables suggested by the SHAP analysis, we conducted an additional exploratory comparison of three model configurations: a clinical-only model, an imaging-only model, and a combined model integrating both feature types. The combined model demonstrated superior predictive performance compared with the clinical-only and imaging-only models (**Supplementary Table 1**), suggesting that imaging features provide complementary diagnostic information beyond clinical biomarkers.

### 3.6 Comparison between model and radiologist diagnoses

To further validate the auxiliary value of the ensemble model in clinical practice, this study compared its performance against that of two radiologists with extensive experience in interpreting DIE MRI studies. The two radiologists independently reviewed the cases in the independent test set in a blinded manner, recording their diagnostic outcomes, which were then compared against the model’s predictions.

**Figure 6:**
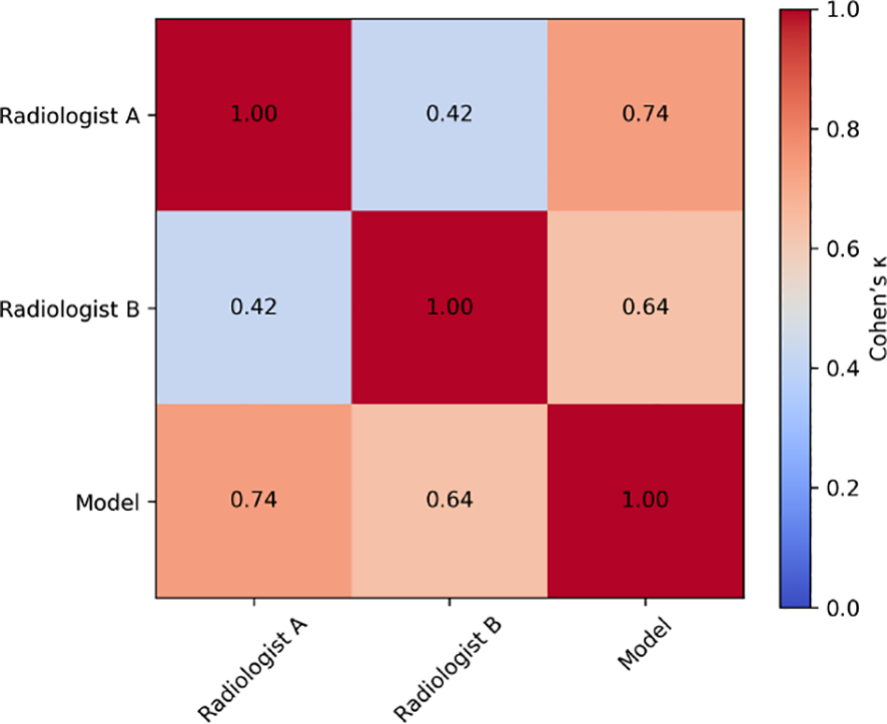
Consistency analysis between radiologists and model diagnoses. The numerical values shown in each cell represent the corresponding Cohen’s kappa coefficients.

Results in **Table 5** and **Figure 6** showed that radiologist A and radiologist B achieved AUCs of 0.824 and 0.813, respectively, with an average radiologist AUC of 0.818 (95% CI: 0.79-0.84), indicating good discriminatory ability for both. The inter-observer agreement coefficient (Cohen’s κ) was 0.42, suggesting moderate consistency between the radiologists. In contrast, the ensemble model performed significantly better on the same test set, achieving an AUC of 0.938 (95% CI: 0.883–0.979), with a sensitivity of 0.920, a specificity of 0.796, an overall accuracy of 0.859, and an F1-score of 0.868. The DeLong’s test indicated a statistically significant difference compared to the average radiologist diagnosis (*P* < 0.05), demonstrating that the model achieved higher discriminatory performance on this dataset.

**Table 5:** Performance comparison between model and radiologists.

Reader/model	AUC (95% CI)	Sensitivity	Specificity	Accuracy	F1-score	PPV	NPV
Radiologist_A	0.824(0.741–0.907)	0.857	0.790	0.824	0.832	0.809	0.842
Radiologist_B	0.813(0.728–0.898)	0.786	0.840	0.812	0.810	0.835	0.791
Average radiologists	0.818(0.735–0.901)	–	–	0.818	–	–	–
Ensemble model	0.938 (0.883-0.979)	0.920	0.796	0.859	0.868	0.918	0.900
AI-assisted radiologists	–	–	–	0.920	–	–	–

AUC, Area under the curve; AI, Artificial intelligence; PPV, Positive predictive value; NPV, Negative predictive value.

Furthermore, simulating an “artificial intelligence (AI)-assisted reading” scenario, where the model’s prediction was provided as auxiliary information to the radiologists for a second assessment, showed that the average diagnostic accuracy of the radiologists increased to 0.920 (an improvement of approximately 10% compared to independent reading), and the average interpretation time decreased by about 30%. This result indicates that the model not only possesses high-precision automated discrimination capability but also effectively enhances radiologists’ diagnostic efficiency and consistency, highlighting its significant potential for clinical application.

## 4 Discussion

In recent years, MRI has played a crucial role in the diagnosis and preoperative assessment of DIE. Recent studies have increasingly demonstrated that MRI-based radiomics and machine learning approaches can improve the detection and characterization of deep infiltrating endometriosis, particularly by capturing subtle imaging heterogeneity beyond conventional visual assessment (Lorusso et al., 2021; VanBuren et al., 2024). However, traditional reading methods remain limited by radiologist experience and subjective image interpretation, often failing to meet clinical demands for diagnostic consistency and sensitivity when faced with complex signal heterogeneity and multifocal lesion distribution. The introduction of radiomics and ML technologies enables the quantitative analysis of high-dimensional imaging features, offering a novel objective assessment tool for complex lesions (Majumder et al., 2024; Schmitz and Sedaghat, 2025). Nonetheless, previous studies have often focused on single-sequence imaging or single algorithms, leaving room for improvement in model stability, calibration, and clinical interpretability (Buvat et al., 2025), and systematic exploration of integrated modeling specifically for DIE, a distinct pathological entity, has been lacking. Addressing this, our study developed and validated an AUC-weighted ensemble ML model based on multi-sequence MRI and clinical parameters, aiming to ensure high discriminatory performance while enhancing model robustness and interpretability. By comparing its performance against individual models and radiologists, we further evaluated the advantages and clinical potential of this AI model in the complex task of pelvic lesion identification.

The ensemble model developed in this study achieved an AUC of 0.938, sensitivity of 0.920, specificity of 0.796, and overall accuracy of 0.859 on the independent test set, outperforming all individual base models. Among the base models, CatBoost and RF both achieved AUCs of 0.936, while XGBoost was slightly lower (AUC 0.930). The ensemble model, formed by AUC-weighted fusion, demonstrated more balanced performance, maintaining high sensitivity while improving calibration and stability. Bootstrap resampling analysis revealed a concentrated AUC distribution with small variance, indicating good robustness of the model under sample perturbation. Compared to previous studies, the discriminatory performance of our model is notable. Li et al. (Li, Li et al., 2022) developed a T2-weighted MRI radiomics model based on ML methods that reliably predicted survival in glioma patients and aided in preoperative assessment of tumor-associated macrophage infiltration; In a multicenter study, Acad Radiol et al (Kang et al., 2025). utilized multiparameter MRI combined with clinical indicators to develop an ML model for predicting treatment response to combination therapy in unresectable hepatocellular carcinoma, achieving an AUC of 0.956 with the combined model, significantly outperforming models based on imaging features alone. This echoes our findings, further underscoring the broad applicability and robustness of multi-modal fusion and ensemble learning strategies in predicting complex diseases. The performance differences observed in our study can likely be attributed to: (1) The ensemble learning strategy effectively integrated the capabilities of different algorithms to characterize non-linear features; (2) The use of whole-image features avoided the subjective bias associated with ROI delineation, enhancing the global representativeness of features; (3) The inclusion of clinical indicators (CA125, CRP, etc.) increased the model’s sensitivity to pathophysiological differences.

Furthermore, calibration curve analysis showed that the ensemble model’s predicted probabilities were closest to the ideal line, with a low Brier score, indicating excellent probability calibration. DCA results also demonstrated that the ensemble model provided the highest net benefit across the commonly used clinical threshold range (0.2-0.8). This further validates the model’s reliability and potential for widespread adoption from a clinical utility perspective. From a clinical perspective, predicted probabilities generated by the model may help stratify patients according to the likelihood of DIE. For example, patients with higher predicted probabilities could be prioritized for further targeted imaging review or surgical planning, whereas those with lower probabilities may benefit from conservative management or additional follow-up. Therefore, the model may serve as a supportive tool to assist clinical decision-making rather than replacing radiologist judgment.

Traditional ML models often suffer from a “black box” effect, limiting their clinical application (Topol, 2019; Keni, 2024). This study is the first to introduce an AUC-weighted SHAP fusion interpretability framework for DIE identification, integrating the contributions of three sub-models (RF, XGBoost, CatBoost) to provide unified global and local explanations. The weighted SHAP analysis identified CRP, CA125, symptom duration, hemoglobin, and BMI as the top five contributing features. CRP and CA125 are biomarkers of inflammation and metabolic activation, and their elevation reflects the activity of ectopic endometrial lesions and the systemic inflammatory response (Chen et al., 2022), consistent with previous reports (Drasin et al., 2023). The significant contribution of symptom duration suggests that long-term chronic inflammation and pelvic fibrotic changes are important in the model’s discrimination. The negative contributions of hemoglobin and BMI hint that chronic blood loss and nutritional status might play protective or modulatory roles in DIE pathological progression (Campara et al., 2025).

From an imaging perspective, features reflecting T2 signal intensity and diffusion restriction also positively influenced the model output, indicating that signal heterogeneity, cellular density, and stromal fibrosis are key imaging indicators. This aligns with the pathological mechanism: DIE lesions are often accompanied by glandular invasion, fibrous tissue proliferation, and chronic inflammatory cell infiltration, leading to local T2 hyperintensity and diffusion restriction (Rousset et al., 2023; Alonzo et al., 2024). Therefore, the high-weight features identified by the model are not only statistically significant but also pathologically plausible. Individual SHAP force plots further revealed that different combinations of features drove the model’s output direction variably for different patients. For instance, high CRP, CA125, and long duration collectively pushed the prediction towards positive, while high hemoglobin and BMI shifted it towards negative. This visual interpretability provides an intuitive link between the model output and clinical understanding for clinicians; however, the SHAP-based interpretations remain statistical explanations and require further biological validation in future studies.

From a clinical perspective, the relatively high contribution of inflammatory biomarkers such as CRP and CA125 may raise the possibility that the model functions primarily as a clinical classifier. To address this concern, we conducted an exploratory comparison of three model configurations, including a clinical-only model, an imaging-only model, and a combined model integrating both feature types. The results showed that the combined model consistently achieved higher predictive performance than either the clinical-only or imaging-only models. This finding suggests that imaging-derived features provide complementary diagnostic information beyond clinical biomarkers and highlights the value of integrating multimodal data for improved diagnostic accuracy.

Whether AI models can surpass or assist human experts in actual diagnosis is a key question in medical AI application. Our results showed that the ensemble model’s AUC (0.938) was significantly higher than the average AUC of the two radiologists (0.818). The inter-radiologist Cohen’s κ was 0.42, indicating moderate agreement, whereas the model showed stable performance within the current internal test set. This suggests that, in this dataset, the AI system may capture complementary patterns across multi-sequence MRI and clinical variables, and may serve as a supportive tool to assist radiologists in complex imaging assessment. In the simulated AI-assisted diagnosis scenario, the radiologists’ average accuracy increased from 0.818 to 0.920, and interpretation time decreased by approximately 30%. This result aligns with previous AI-assisted studies in breast MRI (van Winkel et al., 2021), indicating that AI’s optimal value lies not in replacement but in enhancing interpretation consistency and diagnostic efficiency. For multifocal and heterogeneous conditions such as DIE, AI models may function as intelligent screening and decision-support tools by providing quantitative probability estimates for disease presence, which may assist radiologists in identifying patients with a higher likelihood of DIE and support more consistent diagnostic assessment. However, the comparison involved only two radiologists from a single center, and the results should therefore be interpreted cautiously. Importantly, AI systems should be viewed as decision-support tools rather than replacements for radiologists. It should also be noted that the present framework is a classification-based model rather than a detection or segmentation model; therefore, it does not provide explicit spatial localization of lesions on MRI. Excessive reliance on automated outputs may introduce the risk of automation bias, in which clinicians may over-trust algorithmic predictions. Therefore, maintaining human oversight and integrating AI outputs with clinical judgement remain essential for safe clinical implementation.

Compared to previous studies often based on single-sequence imaging or single-algorithm models, this study achieved innovative progress in three aspects: (1) The whole-image radiomics strategy overcame the subjectivity and reproducibility issues of traditional ROI delineation, allowing features to more comprehensively reflect the overall pelvic signal characteristics; (2) The weighted ensemble learning strategy, using AUC-adaptive weights to fuse the advantages of different algorithms, significantly improved model robustness; (3) The weighted SHAP fusion interpretability framework was used to provide unified global and local explanations for the ensemble model, facilitating clinical interpretation of model outputs; however, these explanations remain statistical and warrant further external and biological validation.

Mechanistically, this improvement stems from the complementary nature of different algorithms in the feature space. RF excels in identifying variable interactions, XGBoost handles sparse and high-dimensional data well, and CatBoost has advantages in categorical variable encoding and non-linear fitting. Weighted fusion allows the model to dynamically balance different feature types, optimizing the overall decision boundary.

Pathologically, the high-weight features prioritized by the model (CRP, CA125, T2 signal) align closely with the “inflammation-fibrosis-vascular change” pathological triad of DIE (Vissers et al., 2024; Yuruk et al., 2025). This consistency suggests that the identified high-weight features are clinically plausible; nevertheless, direct biological validation is required to confirm the underlying mechanisms. In addition, another methodological consideration is the use of a whole-image radiomics strategy rather than lesion-based segmentation. While this approach reduces potential inter-observer variability associated with manual ROI delineation and allows the model to capture global pelvic imaging characteristics, it may also introduce signals from surrounding anatomical structures and non-lesion tissues. Such background information could potentially dilute disease-specific imaging features. Therefore, future studies incorporating precise lesion segmentation or automated localization algorithms may further improve the biological specificity and interpretability of the extracted features.

## 5 Limitations and future directions

This study still has several limitations. First, its single-center retrospective design, while possessing an adequate sample size, limits regional generalizability. Thus, future validation in multi-center prospective cohorts is necessary. In particular, external validation using independent multi-center datasets is needed to confirm model robustness and generalizability. Second, while whole-image radiomic features capture global signal distribution, they may also introduce background information from non-lesion tissues, which could potentially dilute disease-specific imaging signals. Future studies incorporating lesion-level segmentation or automated localization methods may further improve the biological specificity of the extracted features. Third, the model utilized only static imaging sequences and did not incorporate functional imaging data such as dynamic contrast-enhanced MRI or quantitative mapping techniques. Future work could integrate deep learning–derived features and multi-modal fusion strategies to further enhance the model’s spatiotemporal sensitivity and predictive performance. Fourth, the statistical framework involved multiple candidate features during the exploratory screening process. Although regularization techniques such as LASSO with cross-validation were applied to reduce overfitting and false discovery risk, formal multiplicity correction was not performed. Therefore, the selected predictors should be interpreted as exploratory findings that require validation in larger external cohorts. Another consideration is the potential heterogeneity of endometriosis subtypes. In this study, the primary objective was to develop a diagnostic model to distinguish DIE from controls rather than to classify different subtypes of endometriosis. Therefore, subtype-specific analyses were not performed. Future studies with larger multicenter cohorts could further investigate whether radiomic patterns differ across endometriosis subtypes and whether subtype-specific models could improve diagnostic precision.

## 6 Conclusion

This study develops a weighted ensemble ML model based on multimodal MRI and clinical features for the diagnosis of DIE. The model demonstrated strong discriminatory performance and promising stability within the internal independent test set (AUC 0.938) and provided statistical interpretability through weighted SHAP fusion, revealing the core roles of features like CRP, CA125, and T2 signal intensity in the model’s decision-making. The model demonstrated strong discriminatory ability and may serve as a supportive tool for radiologists in DIE diagnosis. Under AI assistance, radiologist accuracy increases to 0.920 and interpretation time decreases by approximately 30%, demonstrating its potential value in clinical auxiliary diagnosis. In summary, the developed interpretable ensemble model combines high efficiency with explainability for the intelligent identification of DIE, suggesting potential clinical utility as an AI-assisted decision support tool; further external validation in multi-center cohorts is required before broader clinical implementation.

## Data Availability

The original contributions presented in the study are included in the article/**Supplementary Material**. Further inquiries can be directed to the corresponding author.
