# Supplementary material for: An ensemble machine learning model based on magnetic resonance imaging features for diagnosing deep infiltrating endometriosis
Source: Front Physiol. 2026 May 29;17:1753847. doi: 10.3389/fphys.2026.1753847 (PMC13259998; doi:10.3389/fphys.2026.1753847)
Supplement: Supplementary file 1 [file Table1.docx]

Supplementary Table S1.Comparative performance of clinical-only, imaging-only, and combined models

| Model | AUC (95% CI) | Sensitivity | Specificity | Accuracy |
| --- | --- | --- | --- | --- |
| Clinical-only model | 0.864 (0.802–0.916) | 0.842 | 0.778 | 0.812 |
| Imaging-only model | 0.821 (0.752–0.883) | 0.795 | 0.761 | 0.782 |
| Combined model | 0.938 (0.892–0.972) | 0.92 | 0.796 | 0.859 |
